# Studies on 3-Oxoalkanenitriles: Novel Rearrangement Reactions Observed in Studies of the Chemistry of 3-Heteroaroyl-3-Oxoalkanenitriles as Novel Routes to 2-Dialkylaminopyridines

**DOI:** 10.3390/molecules17010897

**Published:** 2012-01-18

**Authors:** Hamad M. Al-Matar, Khaled D. Khalil, Mona F. Al-Kanderi, Mohamed H. Elnagdi

**Affiliations:** 1 Chemistry Department, Faculty of Science, University of Kuwait, P.O. Box 5969, Safat 13060, Kuwait; 2 Chemistry Department, Faculty of Science, Cairo University, Giza 12613, Egypt

**Keywords:** cyanoacetylation, dialkylaminopyridine, NOE experiments, 3-Oxoalkanenitrile, X-ray, zeolite

## Abstract

3-Aroyl and 3-heteroaroyl substituted 3-oxoalkanenitriles were synthesized by the reactions of activated aromatic and hetero-aromatic substances with cyanoacetic acid in the presence of acetic anhydride. As part of studies focusing on the preparation of cyanoacetyl-1-*N*-methylbenzimidazole, we observed that reaction of *N*-methyl-benzimidazole with the cyanoanhydride formed by condensation of cyanoacetic acid with acetic anhydride, leads to the formation of 2-(1,3-diacetyl-2,3-dihydro-1*H*-benzo[d]-imidazol-2-yl)acetonitrile (**5**), whose structure was confirmed by X-ray crystallographic analysis. 3-Oxoalkanenitriles **3a**,**b** were observed to undergo condensation reactions with dimethylformamide dimethyl acetal (DMFDMA) to afford the corresponding enamino-nitriles, which react with malononitrile to give 2-dialkylaminopyridines through a pathway involving a new, unexpected rearrangement process. Reactions of 3-oxoalkanenitriles with ethyl acetoacetate were found to afford 2-oxopyran-3-carbonitriles, also occurring via this unexpected rearrangement process. Mechanisms to account for both rearrangement reactions are suggested. In addition, reactions of 3-oxoalkanenitriles with acetylacetone in acetic acid in the presence of ammonium acetate result in the formation of pyridine-3-carbonitriles. Finally, upon heating in the presence of zeolite 3-oxoalkanenitriles **3b**,**c** self-trimerized to produce the corresponding aniline derivatives **23b**,**c**.

## 1. Introduction

In several previous studies, we have explored the chemistry and synthesis of 3-oxoalkanenitriles [1,2,3,4,5]. A somewhat recent approach to these targets via reaction of electron rich aromatic indoles and pyrroles with cyanoacetic anhydride, first reported by Slatt *et al.* [6], has attracted plenty of attention [7,8]. As part of this effort, we recently developed routes for the preparation of 3-aroyl and heteroaroyl substituted 3-oxoalkanenitriles that rely on the use of reactions of electron rich aromatic compounds with cyanoacetic acid promoted by acetic anhydride, either in the presence or absence of catalysts. Because this chemistry enables easy access to this class of compounds, these observations have promoted renewed interest in this area. Earlier [7,8,9,10], we described several novel routes for the synthesis of pyrans and pyridines that begin with 3-oxoalkanenitriles. The current effort has focused on the chemical reactivity and preparation of 3-substituted 3-oxoalkanenitriles and has shown that these substances can be readily generated through a novel cyanoacetylation route described below.

## 2. Results and Discussion

**Scheme 1:**
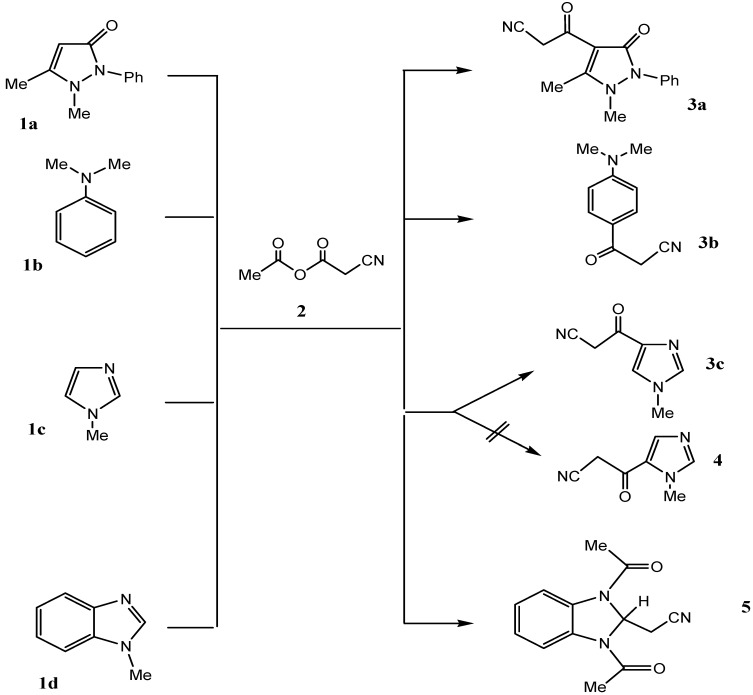
Cyanoacetylation of some π-excessive systems.

**Figure 1:**
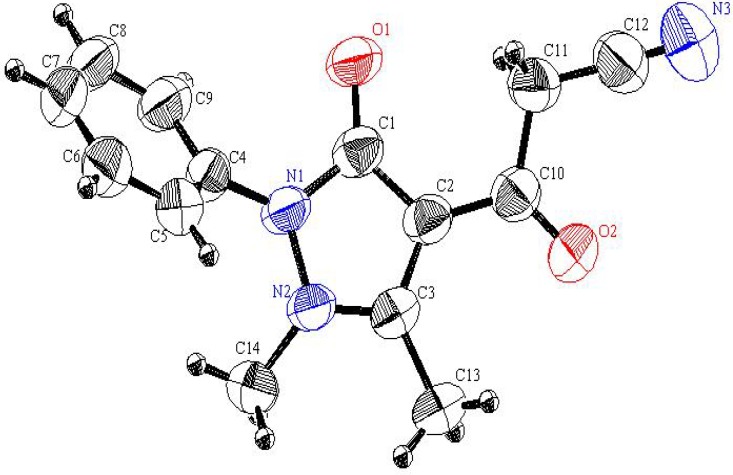
An ORTEP plot of the X-ray crystal structure of **3a**.

In a previous study, we explored potential routes for the preparation of derivatives of the heteroaromatic substituted oxoalkanenitrile **3**, starting with electron rich aromatic compounds **1**. The current efforts were targeted at exploring the potential use of a new rearrangement reaction we observed very recently. We noted that reactions of pyrazolone **1a** and aniline derivative **1b** with the cyanoanhydride **2**, generated by condensation of cyanoacetic acid with acetic anhydride, result in the formation of the corresponding heteroaroyl and aryl substituted 3-oxoalkanenitriles **3a** and **3b** in excellent yields (Scheme 1). The structure of **3a** was assigned by using X-ray crystallographic analysis (Figure 1) (CCDC 2011) [11]. 

In contrast, *N*-methylimidazole **1c** failed to react with **2** under conditions identical to those used in the synthesis of **3a** and **3b**. However these substances do react when InCl_3_ is present in the mixture to afford a product resulting from monocyanoacetylation of **1c**. Preliminary inspection of the analytical data for this product suggested that it could be either one of the regioisomeric products **3c** and **4**. The results of NOE difference experiments, and in particular the fact that irradiation of methyl protons at 2.4 ppm enhanced the intensities of the imidazole CH protons at 7.29 and 8.19 ppm and *vice versa*, revealed a close spacial proximity of the *N*-methyl group and imidazole ring CH and suggested that the structure of the product is best represented by **1c** (Scheme 1).

**Figure 2:**
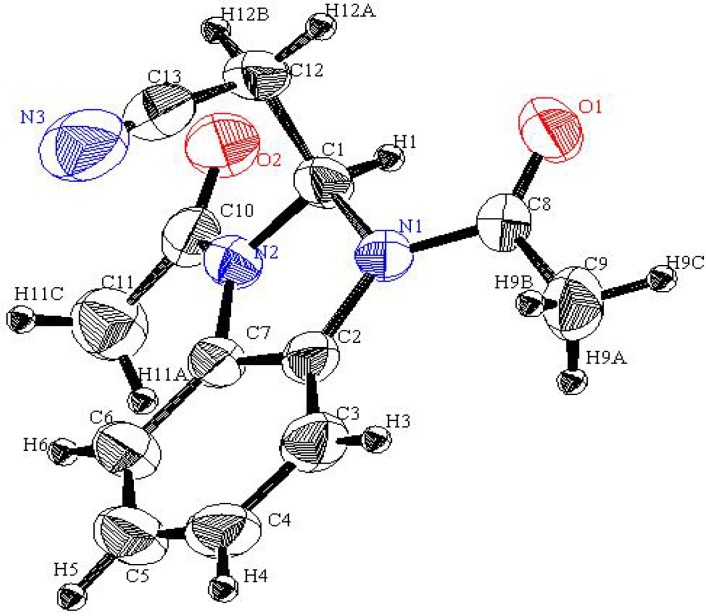
An ORTEP plot of the X-ray crystal structure of **5**.

**Scheme 2:**
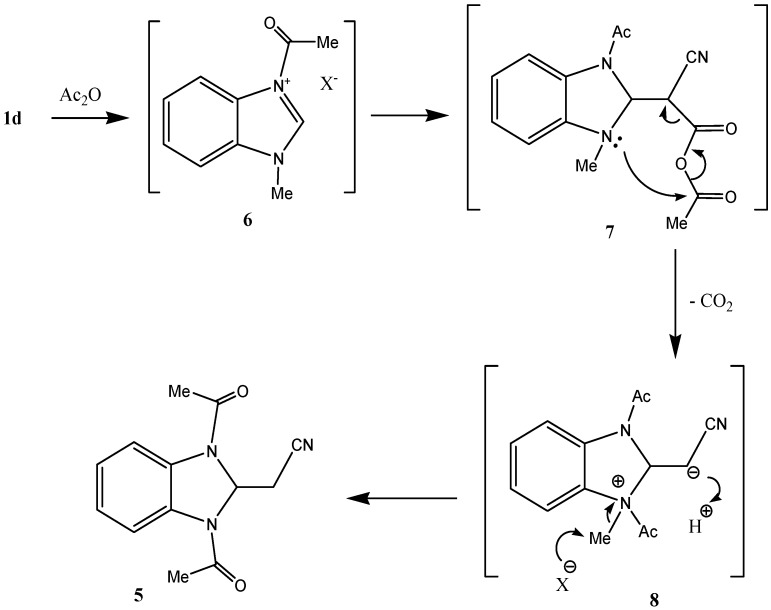
Suggested mechanism for formation of **5**.

Interestingly, reaction of *N*-methylbenzamidazole **1d** with cyanoanhydride **2** results in the formation of an unexpected productwith a molecular formula of C_13_H_13_N_3_O_2_ in 50% yield (Scheme 1). X-ray crystallographic analysis (Figure 2) was used to unambiguously assign the structure of this substance as the unusual bis-acetylbenzimidazolidine **5** (CCDC 2011) [12]. It is believed that **5 **is produced via the mechanistic route displayed in Scheme 2, involving intermediates **6–8**.

**Scheme 3:**
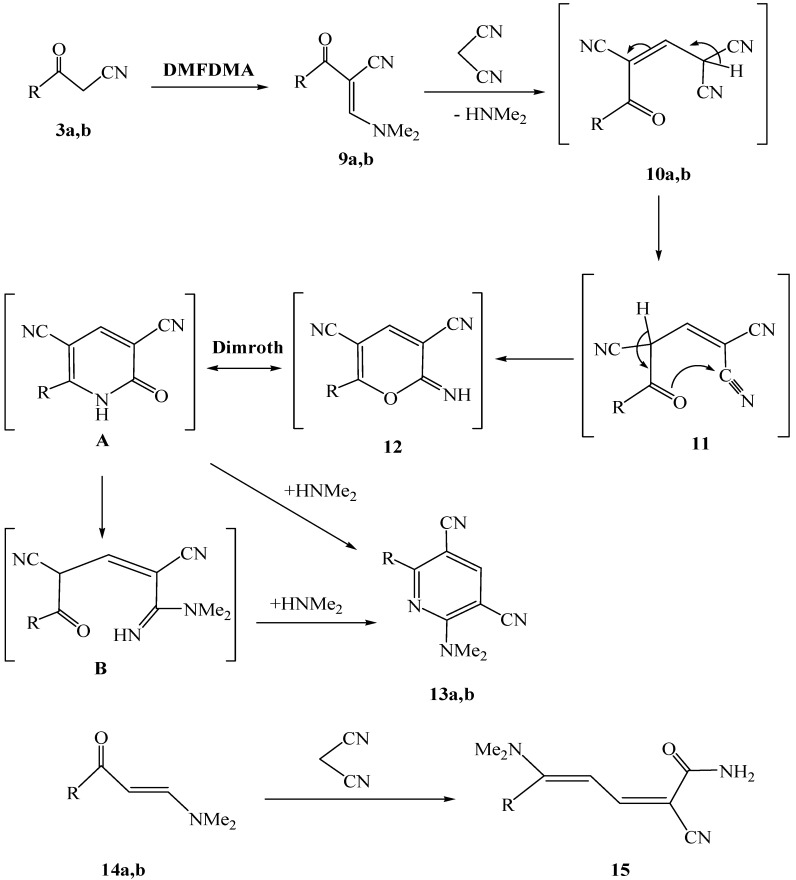
Formation of dialkylaminopyridine derivatives **13a** and **13b**.

The heteroaroyl and aryl substituted 3-oxoalkanenitriles **3a** and **3b** were observed to undergo condensation reactions with dimethylformamide dimethylacetal (DMFDMA) to yield the corresponding enaminonitriles **9a**,**b** (Scheme 3). The products have *trans*-stereochemistry, as indicated by the results of NOE difference experiments that show the close spacial proximity of their respective olefinic methine protons and the methyl protons in **9a** and aryl protons in **9b**.

Further studies demonstrated that the enaminonitriles **9a** and **9b** react with malononitrile in refluxing ethanol containing piperidine to yield the corresponding dialkylaminopyridine-3,5-dicarbonitriles **13a** and **13b** (cf. Scheme 3). 

It is believed that in these processes malononitrile adds to the enaminonitriles to yield intermediate Michael adducts **10a**,**b** that readily cyclize to generate pyranimine **12**, which undergo a Dimroth rearrangement to give pyridone **A**. The latter undergoes ring opening to afford intermediate **B** that then reacts with dimethylamine to yield the observed pyridines **13a** and **13b**. Although to our knowledge a rearrangement reaction of this type has not yet been reported, it involves a sequence of events that parallel those involved in the formation of dieneamide **15** in the reaction of **14** with malononitrile, described earlier by Al-Mousawi [13] and Khalil [14] (Scheme 3). We reported in a recent related work the X-ray crystal structure of 2-(1-methyl-1*H*-pyrrol-2-yl)-6-(piperidin-1-yl)pyridine-3,5-dicarbonitrile, that was prepared by reaction of enaminonitrile of 2-cyanoacetyl-1-methylpyrrole with malononitrile in presence of piperidine, with leaving no doubt about its structure [9]. 

**Scheme 4:**
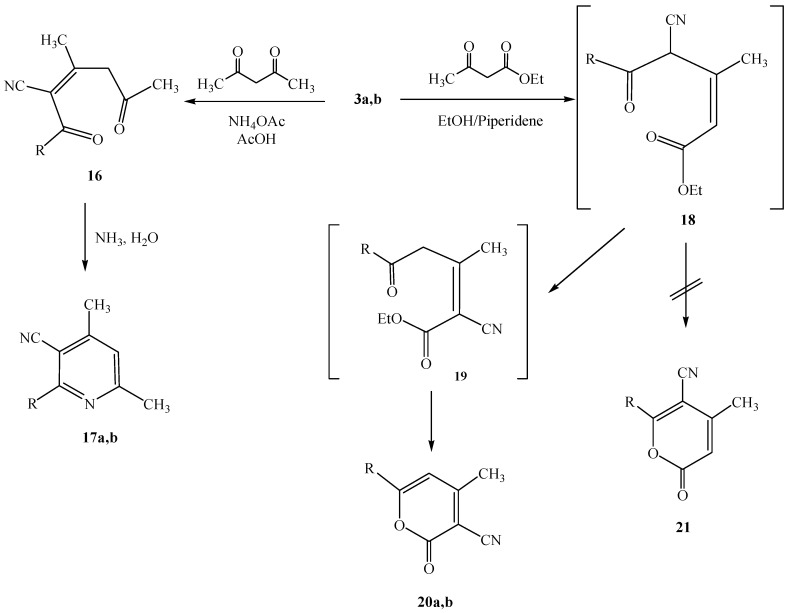
Formation of pyridinecarbonitrile **18a** and **18b** and pyranonecarbonitrile **20a** and **20b**.

Additional studies revealed that heteroaroyl and aryl substituted 3-oxoalkanenitriles **3a** and **3b** condense with acetylacetone in acetic acid in the presence of ammonium acetate to yield the dimethylpyridine derivatives **17a** and **17b** (Scheme 4). These oxoalkanenitriles also react with ethyl acetoacetate via water and ethanol elimination. Although it seemed reasonable that the products formed in these processes would be the pyranones **21** that would result from a bimolecular condensation that generates **18** (Scheme 4), nevertheless, taking into consideration the possibility that a skeletal rearrangement similar to one observed in our recent studies [9] could take place in this process, detailed NMR experiments were performed. Evidence supporting **20a** and **20b** as the structures of these products came from the results of NOE difference experiments, in which irradiation of the pyran ring CH protons at 5.9 ppm enhanced the intensities of the methyl proton resonances at 1.54 ppm. A plausible pathway for the formation of **20a** and **20b** from respective reactions of the heteroaroyl and aryl substituted 3-oxoalkanenitriles **3a** and **3b** involves initial production of intermediate **18** that then undergoes a documented [15,16,17] 1,3-cyano shift to yield **19**. Cyclization of **19** then yields **20a** and **20b**.

**Scheme 5:**
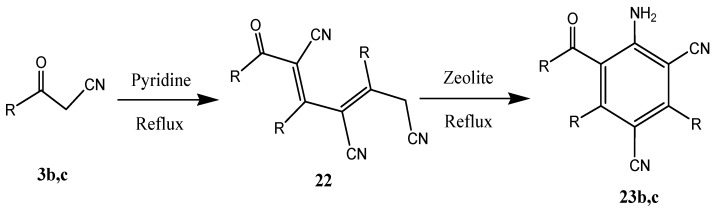
Self-condensation (trimerization) reaction of oxoalkanenitriles **3b** and **3c**.

Further efforts showed that 3-oxoalkanenitriles **3b,c** can be utilized as precursors for quinolinones. By employing a modification of the self-condensation reaction conditions reported earlier by Elnagdi *et al.* [18] (Scheme 5) and Breil *et al*. [19], heating **3b** and **3c** over activated zeolite was found to lead to formation of the corresponding arenes **23b** and **23c** via a route possiblly involving the intermediacy of **22**.

**Scheme 6:**
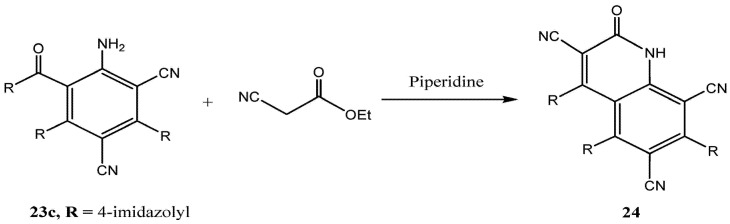
Synthesis of quinolinone derivative **24**.

Finally, we observed that the aniline derivatives **23c** (Scheme 6) readily participate in condensation reactions with ethyl cyanoacetate in ethanolic piperidine to yield the corresponding quinolinones **24** (Scheme 6).

## 3. Experimental

### 3.1. General

Melting points were recorded on Gallenkamp apparatus and are reported uncorrected. Infrared spectra (KBr) were determined on a Perkin-Elmer 2000 FT-IR system. NMR measurements were determined using a Bruker DPX spectrometer, at 600 MHz for ^1^H-NMR and 125 MHz for ^13^C-NMR, in DMSO-*d*_6_ as solvent and using TMS as internal standard. Mass spectra were measured on MS 30 and MS 9 (AEI) spectrometers, with EI 70 eV. All fine chemicals and organic solvents were purchased from Aldrich Chemicals and used without any further purification. Also, zeolite (CAS Number: 1318-02-1) was purchased from Aldrich Chemicals. Elemental analyses were measured on a LECO CHNS-932 Elemental Analyzer. Copies of original data can be provided on request.

### 3.2. Synthesis of Acetic 2-Cyanoacetic Anhydride (**2**)

A mixture of acetic anhydride (1.02 g, 10.0 mmol) and (0.85 g, 10.0 mmol) of cyanoacetic acid in dry dioxane (15 mL) was stirred at reflux for 15 min. The formed mixture was filtered and the filtrate was used after cooling as the cyanoacetylating agent **2** for reactions of aromatic substrates in the presence of a catalytic amount (10% wt.) of InCl_3_ as a Lewis acid.

### 3.3. Cyanoacetylation Reactions of **1a–d** with Acetic 2-Cyanoacetic Anhydride (**2**)

A mixture of pre-prepared acetic 2-cyanoacetic anhydride (**2**, 1.27 g, 10.0 mmol), the starting aromatic or heteroaromatic compound (10.0 mmol), and indium trichloride (0.12 g, 10% wt.) in case of 1-methylimidazole (**1c**) and 1-methylbenzimidazole (**1d**), in dry dioxane (15 mL) was stirred at reflux for 1 h. The reaction mixture was then poured into water. The formed solid product was then collected by filtration, washed with water and crystallized from ethanol. Cyanoacetylation of benzimidazole **1d** afforded one isolable products **5** as the major product. The product was isolated by column chromatography using ethyl acetate-*n*-hexane (1:3) as eluent and its structure confirmed by X-ray determination.

*3-(1,5-Dimethyl-3-oxo-2-phenyl-2,3-dihydro-1*H*-pyrazol-4-yl)-3-oxopropanenitrile* (**3a**). Yellow crystals (85%, m.p. 156 °C); IR (KBr): *υ* = 2187 (CN), 1705, 1685 (two C = O) cm^−1^, ^1^H-NMR: *δ* = 2.62 (s, 3H, CH_3_), 3.36 (s, 3H, CH_3_), 4.32 (s, 2H, CH_2_), 7.36–7.59 (m, 5H, phenyl); ^13^C NMR: *δ* = 30.5 (CH_3_), 33.4 (CH_3_), 38.8 (CH_2_), 116.2 (CN), 101.8, 127.5, 128.9, 129.4, 133.2, 153.9 (aromatic carbons), 162.7 (amide C=O), 182.4 (ketonic C=O); MS, *m/z* (%), 255.1 (M^+^, 43), 215.1 (100%). Anal. Calcd. for C_14_H_13_N_3_O_2_: C, 65.87; H, 5.13; N, 16.46. Found: C, 65.79; H, 5.02; N, 16.34.

*3-(4-(Dimethylamino)phenyl)-3-oxopropanenitrile* (**3b**). Dark brown solid (90%, m.p. 164 °C); IR (KBr): *υ* = 2182 (CN), 1700 (C = O) cm^−1^, ^1^H-NMR: *δ* = 3.03 (s, 6H, two CH_3_), 4.55 (s, 2H, CH_2_), 7.21 (d, 2H, *J* = 8 Hz, benzene), 7.75 (d, 2H, *J* = 8 Hz, benzene); ^13^C-NMR: *δ* = 29.8 (Two CH_3_), 39.5 (CH_2_), 110.6, 115.9 (CN), 129.4, 130.5, 153.8 (aromatic carbons), 186.2 (C=O); MS, *m/z* (%), 188.1 (M^+^, 43), 148.1 (100%). Anal. Calcd. for C_11_H_12_N_2_O: C, 70.19; H, 6.43; N, 14.88. Found: C, 70.04; H, 6.28; N, 14.59.

*3-(1-Methyl-1*H*-imidazol-4-yl)-3-oxo-propionitrile* (**3c**). Brown solid (65%); IR (KBr): *υ* = 2180 (CN), 1704 (C = O) cm^−1^, ^1^H-NMR: *δ* = 2.46 (s, 3H, CH_3_), 4.27 (s, 2H, CH_2_), 7.29 (s, 1H, C-4 imidazole), 8.19 (s, 1H, C-2 imidazole); ^13^C-NMR: *δ* = 33.7 (CH_2_), 35.4 (CH_3_), 116.7 (CN), 109.6, 127.6, 133.3 (imidazole carbons), 183.3 (C=O); MS, *m/z* (%), 149.1 (M^+^, 43), 109.1 (100%). Anal. Calcd. for C_7_H_7_N_3_O: C, 56.37; H, 4.73; N, 28.17. Found: C, 56.11; H, 4.65; N, 27.76.

*(1,3-Diacetyl-2,3-dihydro-1*H*-benzoimidazol-2-yl)-acetonitrile* (**5**). Brown solid (50%); IR (KBr): *υ* = 2188 (CN), 1664, 1660 (C = O) cm^−1^, ^1^H-NMR (DMSO-d_6_): *δ* = 2.71 (s, 6H, two CH_3_), 4.13 (s, 2H, CH_2_), 4.89 (s, 1H, C-2 imidazole), 7.11–7.64 (m, 4H, benzene ring); ^13^C-NMR (DMSO-d_6_): *δ* = 30.9 (CH_2_), 34.6 (2 × CH_3_), 38.1 (CH imidazole), 117.2 (CN), 122.6, 126.3, 136.0 (benzene carbons), 168.8 (two C=O); MS, *m/z* (%), 243.1 (M^+^, 18), 157.1 (100%). Anal. Calcd. for C_13_H_13_N_3_O_2_: C, 64.19; H, 5.39; N, 17.27. Found: C, 64.13; H, 5.35; N, 17.02.

### 3.4. Reaction of Cyanoacetyl Derivatives **3a,b** with DMFDMA

A mixture of **3a** or **3b** (10 mmol) and (DMFDMA) (15 mmol) was dissolved in dry xylene (50 mL) and the reaction mixture was refluxed while the reaction was followed to completion by TLC, using ethyl acetate-*n*-hexane (1:3) as eluent. The reaction mixture was concentrated under reduced pressure, cooled and the solid product, so formed, was then filtered and recrystallized from ethanol.

*2-(1,5-Dimethyl-3-oxo-2-phenyl-2,3-dihydro-1*H*-pyrazole-4-carbonyl)-3-(dimethylamino)acrylonitrile* (**9a**). Yellow solid (65%, m.p. 149 °C); IR (KBr): *υ* = 2180 (CN), 1690, 1682 (two C = O) cm^−1^; ^1^H-NMR: *δ* = 1.84 (s, 3H, CH_3_), 1.98 (s, 3H, CH_3_), 2.62 (s, 6H, two CH_3_), 6.83–7.42 (m, 5H, phenyl), 7.86 (s, 1H, enamine); ^13^C-NMR: *δ* = 31.8 (CH_3_), 32.1(CH_3_), 33.6 (Two CH_3_), 116.7 (CN), 96.0, 112.6, 122.4, 124.1, 127.9, 130.9, 136.5, 144.3 (aromatic carbons), 159.2 (amide C=O), 176.8 (ketonic C=O); MS, *m/z* (%), 310.1 (M^+^, 16), 215.1 (100). Anal. Calcd. for C_17_H_18_N_4_O_2_: C, 65.79; H, 5.85; N, 18.05. Found: C, 65.71; H, 5.62; N, 17.84.

*3-(Dimethylamino)-2-(4-(dimethylamino)benzoyl)acrylonitrile* (**9b**). Brown solid (60%, m.p. 206 °C); IR (KBr): *υ* = 2173 (CN), 1695 (C = O) cm^−1^; ^1^H-NMR: *δ* = 2.78 (s, 6H, two CH_3_), 2.90 (s, 6H, two CH_3_), 7.18 (d, 2H, *J* = 8 H*z*, phenyl), 7.72 (d, 2H, *J* = 8 H*z*, phenyl), 8.28 (s, 1H, enamine); ^13^C-NMR: *δ* = 31.7 (CH_3_), 34.0 (CH_3_), 37.6 (Two CH_3_), 116.3 (CN), 119.1, 126.0, 129.1, 131.2, 139.5, 144.3 (aromatic carbons), 183.2 (ketonic C=O); MS, *m/z* (%), 243.1 (M^+^, 16), 148.1 (100). Anal. Calcd. for C_14_H_17_N_3_O: C, 69.11; H, 7.04; N, 17.27. Found: C, 69.06; H, 6.99; N, 17.21.

### 3.5. Reaction of **9a,b** with Malononitrile

A mixture of **9a** or **9b** (10 mmol) and malononitrile (0.66 g, 10 mmol) was dissolved in absolute ethanol (30 mL) and the reaction mixture was refluxed for 6 h, then concentrated under reduced pressure, cooled and the solid product, so formed, was then filtered and recrystallized from ethanol.

*2-(1,5-Dimethyl-3-oxo-2-phenyl-2,3-dihydro-1*H*-pyrazol-4-yl)-6-(dimethylamino)pyridine-3,5-dicarbonitrile* (**13a**). Yellow solid (60%, m.p. 172 °C); IR (KBr): *υ* = 2185, 2175 (two CN), 1665 (C=O) cm^−1^; ^1^H-NMR: *δ* = 2.45 (s, 3H, CH_3_), 2.61 (s, 3H, CH_3_), 2.84 (s, 6H, two CH_3_), 7.10–7.51 (m, 5H, phenyl), 8.48 (s, 1H, pyridine); ^13^C-NMR: *δ* = 29.7 (two CH_3_), 32.4 (two CH_3_), 116.4 (two CN), 94.5, 104.5, 126.9, 131.1, 135.2, 141.6, 143.1, 159.5, 162.6 (aromatic carbons), 169.8 (C=O); MS, *m/z* (%), 358.1 (M^+^, 16), 177.1 (100). Anal. Calcd. for C_20_H_18_N_6_O: C, 67.02; H, 5.06; N, 23.45. Found: C, 66.95; H, 4.98; N, 23.36. 

*2-(Dimethylamino)-6-(4-(dimethylamino)phenyl)pyridine-3,5-dicarbonitrile* (**13b**). Dark brown solid (65%, m.p. 137 °C); IR (KBr): *υ* = 2190, 2182 (CN) cm^−1^; ^1^H-NMR: *δ* = 2.28 (s, 6H, two CH_3_), 2.98 (s, 6H, CH_3_), 2.90 (s, 6H, two CH_3_), 7.04 (d, 2H, *J* = 8 H*z*, phenyl), 7.76 (d, 2H, *J* = 8 H*z*, phenyl), 8.78 (s, 1H, pyridine); ^13^C-NMR: *δ* = 30.6 (2 × CH_3_), 32.4 (2 × CH_3_), 117.1, 117.6 (2 × CN), 122.2, 124.6, 129.6, 135.2, 137.7, 148.4, 149.7, 152.4, 168.3 (aromatic carbons); MS, *m/z* (%), 291.2 (M^+^, 16), 120.1 (100). Anal. Calcd. for C_17_H_17_N_5_: C, 70.08; H, 5.88; N, 24.04. Found: C, 69.94; H, 5.81; N, 23.93.

### 3.6. Reaction of **3a,b** with Acetylacetone

A mixture of **3a** or **3b** (10 mmol), ammonium acetate (1.54 g, 20 mmol), and acetylacetone (1.00 g, 10 mmol) was dissolved in glacial acetic acid (20 mL) and the reaction mixture was refluxed for 24 h, then poured over an ice-water mixture and the solid product, so formed, was then filtered and recrystallized from ethanol.

*2-(1,5-Dimethyl-3-oxo-2-phenyl-2,3-dihydro-1*H*-pyrazol-4-yl)-4,6-dimethylnicotinonitrile* (**17a**). Yellow solid (68%, m.p. 163 °C); IR (KBr): *υ* = 2181 (CN), 1660 (C=O) cm^−1^; ^1^H-NMR: *δ* = 2.87 (s, 6H, two CH_3_), 3.15 (s, 6H, two CH_3_), 6.88–7.29 (m, 5H, phenyl), 8.24 (s, 1H, pyridine); ^13^C-NMR: *δ* = 35.8 (CH_3_), 37.2 (CH_3_), 47.1 (CH_3_), 117.1 (CN), 122.7, 128.9, 131.6, 132.3, 134.6, 150.2, 153.9, 155.0, 157.6 (aromatic carbons), 163.0 (C=O); MS, *m/z* (%), 318.1 (M^+^, 16), 215.1 (100). Anal. Calcd. for C_19_H_18_N_4_O: C, 71.68; H, 5.70; N, 17.60. Found: C, 71.54; H, 5.59; N, 17.28. 

*2-(4-(Dimethylamino)phenyl)-4,6-dimethylnicotinonitrile* (**17b**). Dark brown solid (55%, m.p. 119 °C); IR (KBr): *υ* = 2176 (CN) cm^−1^; ^1^H-NMR: *δ* = 1.56 (s, 6H, two CH_3_), 2.12 (s, 3H, CH_3_), 2.28 (s, 3H, CH_3_), 6.73 (d, 2H, *J* = 8 H*z*, phenyl), 7.84 (d, 2H, *J* = 8 H*z*, phenyl), 8.34 (s, 1H, pyridine); ^13^C-NMR: *δ* = 34.7 (two CH_3_), 39.1 (CH_3_), 116.3 (CN), 104.6, 111.8, 121.7, 125.2, 131.6, 139.1, 146.7, 148.0, 158.2 (aromatic carbons); MS, *m/z* (%), 251.2 (M^+^, 16), 120.1 (100). Anal. Calcd. for C_16_H_17_N_3_: C, 76.46; H, 6.82; N, 16.72. Found: C, 76.13; H, 6.69; N, 16.52.

### 3.7. Reaction of **3a,b** with Ethyl Acetoacetate

A mixture of **3a** or **3b** (10 mmol) and ethyl acetoacetate (1.30 g, 10 mmol) was dissolved in absolute ethanol (20 mL) in the presence of few drops of piperidine as catalyst. The reaction mixture was refluxed for 8 h, then concentrated under reduced pressure and the solid product, so formed, was then filtered and recrystallized from ethanol.

*6-(2,3-Dimethyl-5-oxo-1-phenyl-2,5-dihydro-1*H*-pyrazol-4-yl)-4-methyl-2-oxo-2H-pyran-3-carbonitrile* (**20a**). Yellow solid (58%, m.p. 141 °C); IR (KBr): *υ* = 2215 (CN), 1718, 1668 (C = O) cm^−1^; ^1^H-NMR: *δ* = 1.54 (s, 6H, two CH_3_), 2.51 (s, 6H, 2 × CH_3_), 5.98 (s, 1H, pyran), 6.74–7.77 (m, 5H, phenyl); ^13^C-NMR: *δ* = 34.1 (CH_3_), 35.2 (CH_3_), 116.2 (CN), 87.5, 104.5, 112.8, 115.1 (pyran), 125.9, 131.4, 135.2, 142.9, 150.2, 154.5 (aromatic carbons) 159.5 (C=O, antipyrine), 162.6 (C=O, pyran); MS, *m/z* (%), 321.1 (M^+^, 16), 215.1 (100). Anal. Calcd. for C_18_H_15_N_3_O_3_: C, 67.28; H, 4.71; N, 13.08. Found: C, 67.13; H, 4.66; N, 12.91. 

*6-(4-(Dimethylamino)phenyl)-4-methyl-2-oxo-2*H*-pyran-3-carbonitrile* (**20b**). Brown solid (50%, m.p. 157 °C); IR (KBr): *υ* = 2185 (CN) cm^−1^; ^1^H-NMR: *δ* = 2.66 (s, 6H, two CH_3_), 3.37 (s, 3H, CH_3_), 5.97 (s, 1H, pyran), 6.52 (d, 2H, *J* = 8 H*z*, phenyl), 7.01 (d, 2H, *J* = 8 H*z*, phenyl); ^13^C-NMR: *δ* = 28.5 (2 × CH_3_), 33.4 (CH_3_), 117.1 (CN), 99.8, 103.6, 111.7, 115.5, 128.8, 134.2, 147.2, 149.9 (aromatic carbons), 162.4 (C=O); MS, *m/z* (%), 254.1 (M^+^, 16), 120.1 (100). Anal. Calcd. for C_15_H_14_N_2_O_2_: C, 70.85; H, 5.55; N, 11.02. Found: C, 70.59; H, 5.32; N, 10.87.

### 3.8. Trimerization of 3-Oxoalkanenitriles **3b,c**

The proper 3-oxoalkanenitrile (**3b** or **3c**, 10 mmol) was refluxed for 8 h in pyridine (20 mL). The reaction mixture was poured over HCl/H_2_O to neutralize the pyridine. The crude solid product was filtered and dried. The product **22** was dissolved in dry dioxane (15 mL) and refluxed again in the presence of a catalytic amount of zeolite (0.2 g, 20% wt.) for 12 h. The crude product was filtered, purified by DMF-EtOH to yield **23**.

*4-Amino-2,6-bis(4-(dimethylamino)phenyl)-5-(4-(dimethylamino)phenyl-4-carbonyl)-isophthalonitrile* (**23b**). Dark brown solid (60%); IR (KBr): *υ* = 3350, 3330 (broad band, NH_2_), 2196, 2185 (CN), 1690 (C=O) cm^−1^; ^1^H-NMR: *δ* = 2.23 (s, 12H, CH_3_), 2.37 (s, 3H, CH_3_), 3.14 (s, 3H, two CH_3_), 6.24 (s, 2H, NH_2_), 6.84–7.61 (m, 12H, phenyl rings aromatic H); ^13^C-NMR: *δ* = 32.7, 35.1, 38.3 (six CH_3_), 116.3, 116.5 (2 × CN), 102.9, 110.2, 119.9, 122.0, 122.8, 127.2, 128.6, 135.0, 140.9, 146.2, 149.1, 151.2, 162.7, 164.3 (aromatic carbons), 181.2 (C=O); MS, *m/z* (%), 411.1 (M^+^, 18), 330.1 (100). Anal. Calcd. for C_33_H_32_N_6_O: C, 74.98; H, 6.10; N, 15.90. Found: C, 74.82; H, 5.93; N, 15.78.

*4-Amino-2,6-bis(1-methyl-1H-imidazol-4-yl)-5-(1-methyl-1*H*-imidazole-4-carbonyl)isophthalonitrile* (**23c**)*.* Pale brown solid (51%); IR (KBr): *υ* = 3340, 3320 (broad band, NH_2_), 2187, 2176 (CN), 1658 (C=O) cm^−1^, ^1^H-NMR: *δ* = 2.91 (s, 3H, CH_3_), 3.24 (s, 6H, 2 × CH_3_), 5.81 (s, 2H, NH_2_), 7.41–7.90 (m, 6H, aromatic H, imidazole); ^13^C-NMR: *δ* = 33.7, 33.9 (three CH_3_), 116.8, 117.5 (2 × CN), 91.3, 104.2, 123.8, 124.1, 128.3, 133.0, 141.3, 145.9, 148.7, 148.9, 163.9, 167.5 (aromatic carbons), 183.7 (C=O); MS, *m/z* (%), 411.1 (M^+^, 18), 330.1 (100). Anal. Calcd. for C_21_H_17_N_9_O: C, 61.31; H, 4.16; N, 30.64. Found: C, 61.14; H, 4.08; N, 30.39.

### 3.9. Condensation Reaction of **23c** with Ethyl Cyanoacetate

A mixture of **23c** (10.0 mmol) and ethyl cyanoacetate (1.13 g, 10 mmol) in absolute ethanol (25 mL) containing a catalytic amount of piperidine was stirred at reflux for 24 h. The reaction mixture was cooled and the product was collected by filtration, and crystallized from ethanol to afford *4,5,7-tris(1-methyl-1*H*-imidazol-4-yl)-2-oxo-1,2-dihydroquinoline-3,6,8-tricarbonitrile* (**24**). Dark brown solid (40%); IR (KBr): *υ* = 3335 (NH), 2181, 2198 (CN), 1656 (C=O) cm^−1^, ^1^H-NMR: *δ* = 2.89 (s, 6H, 2 × CH_3_), 3.13 (s, 3H, CH_3_), 7.13–7.78 (m, 6H, aromatic H, pyrrole), 8.22 (s, 2H, NH); ^13^C-NMR: *δ* = 31.1, 38.2 (three CH_3_), 116.9, 117.4 (2 × CN), 87.3, 105.4, 113.4, 115.0, 120.8, 123.2, 125.9, 128.5, 133.7, 136.2, 139.4, 141.6 (aromatic carbons), 164.1 (C=O); MS, *m/z* (%), 459.8 (M^+^, 18), 81.1 (100%). Anal. Calcd. for C_24_H_16_N_10_O: C, 62.60; H, 3.50; N, 30.42. Found: C, 62.58; H, 3.48; N, 30.37.

## 4. Conclusions

Several novel rearrangement reactions have been observed and plausible mechanisms for these processes presented. Investigations of the scope and limitations of these reactions are underway.

## Supplementary Materials

Supplementary materials can be accessed at: http://www.mdpi.com/1420-3049/17/1/897/s1.
