# Supplementary material for: Studies on 3-Oxoalkanenitriles: Novel Rearrangement Reactions Observed in Studies of the Chemistry of 3-Heteroaroyl-3-Oxoalkanenitriles as Novel Routes to 2-Dialkylaminopyridines
Source: Molecules. 2012 Jan 18;17(1):897–909. doi: 10.3390/molecules17010897 (PMC6268551; doi:10.3390/molecules17010897)
Supplement: Supplementary file 1 [file molecules-17-00897-s001.zip › molecules-12690-supplementary/X Ray Data 1a and 5/X ray data for compound 1a CAAP/Report 1/X-ray Structure Report.rtf]

X-ray Structure Report for CAP220311 
April 10, 2011 
Experimental 
Data Collection
An orange block crystal of C14H13N3O2 having approximate dimensions of 0.200 × 0.200 × 0.200 mm was mounted on a glass fiber. All measurements were made on a diffractometer Mo-Ka radiation.
Cell constants and an orientation matrix for data collection, obtained from a least-squares refinement using the setting angles of 7830 carefully centered reflections in the range 6.44 < 2q < 54.97° corresponded to a primitive orthorhombic cell with dimensions:
a = 12.6554(3) Å
b = 12.5337(3) Å
c = 8.3171(2) Å
V = 1319.25(6) Å3
For Z = 4 and F.W. = 255.28, the calculated density is 1.285 g/cm3. Based on the reflection conditions of:
0kl: k + l = 2n
h0l: h = 2n
packing considerations, a statistical analysis of intensity distribution, and the successful solution and refinement of the structure, the space group was determined to be:
Pna21 (#33)
The data were collected at a temperature of 0 ± 1 °C using the w-2q scan technique to a maximum 2q value of 55.0°. Omega scans of several intense reflections, made prior to data collection, had an average width at half-height of 0.00° with a take-off angle of 6.0°. Scans of (0.00 + 0.00 tan q)° were made at a speed of 0.0°/min (in w).
Data Reduction
Of the 12575 reflections that were collected, 3010 were unique (Rint = 0.1339). No decay correction was applied.
The linear absorption coefficient, m, for Mo-Ka radiation is 0.887 cm−1. The data were corrected for Lorentz and polarization effects.
Structure Solution and Refinement
The structure was solved by direct methods and expanded using Fourier techniques. The non-hydrogen atoms were refined anisotropically. Hydrogen atoms were refined using the riding model. The final cycle of full-matrix least-squares refinement on F2 was based on 3010 observed reflections and 172 variable parameters and converged (largest parameter shift was 0.00 times its esd) with unweighted and weighted agreement factors of: 
R1 = S ||Fo| − |Fc|| / S |Fo| = 0.0627
wR2 = [S(w (Fo2 − Fc2) 2 )/ S w(Fo2)2]1/2 = 0.1893
The standard deviation of an observation of unit weight was 0.98. Unit weights were used. The maximum and minimum peaks on the final difference Fourier map corresponded to 0.39 and s−0.42 e−/Å3, respectively. The absolute structure was deduced based on Flack parameter, −2(2), using 1396 Friedel pairs. 
Neutral atom scattering factors were taken from Cromer and Waber. Anomalous dispersion effects were included in Fcalc; the values for Df' and Df" were those of Creagh and McAuley. The values for the mass attenuation coefficients are those of Creagh and Hubbell. All calculations were performed using the CrystalStructure crystallographic software package except for refinement, which was performed using SHELXL-97.
Experimental Details 
A. Crystal Data 
Empirical Formula	C14H13N3O2	
Formula Weight	255.28	
Crystal Color, Habit	orange, block	
Crystal Dimensions	0.200 × 0.200 × 0.200 mm	
Crystal System	orthorhombic	
Lattice Type	Primitive	
No. of Reflections Used for Unit
Cell Determination (2q range)	7830 ( 6.4–55.0°)	
Omega Scan Peak Widthat Half-height	0.00°	
Lattice Parameters	a = 12.6554(3) Å
b = 12.5337(3) Å
c = 8.3171(2) Å
V = 1319.25(6) Å3	
Space Group	Pna21 (#33)	
Z value	4	
Dcalc	1.285 g/cm3	
F000	536.00	
m(MoKa)	0.887 cm−1	


B. Intensity Measurements 
Diffractometer		
Radiation	MoKa (l = 0.71075 Å)	
Take-off Angle	2.8°	
Detector Aperture	2.0–2.5 mm horizontal
2.0 mm vertical	
Crystal to Detector Distance	21 mm	
Voltage, Current	50kV, 24mA	
Temperature	0.0 °C	
Scan Type	w-2q	
Scan Rate	0.0o/min (in w) (up to 0 scans)	
Scan Width	(0.00 + 0.00 tan q)°	
2qmax	55.0°	
No. of Reflections Measured	Total: 12575
Unique: 3010 (Rint = 0.1339)
Friedel pairs: 1396	
Corrections	Lorentz-polarization	
C. Structure Solution and Refinement 
Structure Solution	Direct Methods (SHELX97)	
Refinement	Full-matrix least-squares on F2	
Function Minimized	S w (Fo2 − Fc2) 2	
Least Squares Weights	w = 1/ [s2(Fo2) + (0.0791P)2 + 0.0000P]
where P = (Max(Fo2,0) + 2Fc2)/3	
2qmax cutoff	55.0°	
Anomalous Dispersion	All non-hydrogen atoms	
No. Observations (All reflections)	3010	
No. Variables	172	
Reflection/Parameter Ratio	17.50	
Residuals: R1 (I > 2.00s(I))	0.0627	
Residuals: R (All reflections)	0.0899	
Residuals: wR2 (All reflections)	0.1893	
Goodness of Fit Indicator	0.985	
Flack Parameter	−2(2)	
Max Shift/Error in Final Cycle	0.000	
Maximum peak in Final Diff. Map	0.39 e−/Å3	
Minimum peak in Final Diff. Map	−0.42 e−/Å3	

Table 1. Atomic coordinates and Biso/Beq.
atom	x	y	z	Beq	
O1	0.8210(2)	0.40075(13)	0.5208(4)	6.40(6)	
O2	0.6338(2)	0.6885(2)	0.5889(4)	6.62(6)	
N1	0.9375(2)	0.5222(2)	0.4110(3)	4.76(5)	
N2	0.9419(2)	0.6341(2)	0.4013(3)	4.57(4)	
N3	0.4441(3)	0.5228(4)	0.7516(8)	10.52(13)	
C1	0.8413(3)	0.4935(2)	0.4831(4)	4.77(5)	
C2	0.7845(2)	0.5923(2)	0.5040(4)	4.59(5)	
C3	0.8492(2)	0.6742(2)	0.4491(4)	4.68(5)	
C4	1.0320(2)	0.4616(2)	0.4254(4)	4.61(5)	
C5	1.1174(3)	0.5012(2)	0.5110(4)	5.28(6)	
C6	1.2066(3)	0.4385(3)	0.5335(5)	6.42(8)	
C7	1.2099(3)	0.3377(3)	0.4690(6)	7.02(9)	
C8	1.1249(3)	0.2977(3)	0.3841(5)	6.78(9)	
C9	1.0351(3)	0.3599(2)	0.3589(4)	5.79(7)	
C10	0.6812(3)	0.6044(2)	0.5757(4)	4.99(5)	
C11	0.6320(3)	0.5021(3)	0.6402(6)	6.37(8)	
C12	0.5274(3)	0.5148(3)	0.7002(6)	7.10(9)	
C13	0.8276(3)	0.7911(3)	0.4384(6)	6.66(8)	
C14	1.0163(3)	0.6849(2)	0.2927(4)	5.35(6)	
Beq = 8/3 p2(U11(aa*)2 + U22(bb*)2 + U33(cc*)2 + 2U12(aa*bb*)cos g + 2U13(aa*cc*)cos b + 2U23(bb*cc*)cos a).
Table 2. Atomic coordinates and Biso involving hydrogen atoms
atom	x	y	z	Biso	
H5	1.1149	0.5698	0.5535	6.34	
H6	1.2638	0.4646	0.5918	7.71	
H7	1.2700	0.2959	0.4826	8.43	
H8	1.1276	0.2286	0.3435	 8.13	
H9	0.9787	0.3340	0.2990	6.94	
H11A	0.6310	0.4494	0.5547	7.64	
H11B	0.6763	0.4745	0.7257	7.64	
H13A	0.8929	0.8289	0.4249	8.00	
H13B	0.7935	0.8146	0.5352	8.00	
H13C 	0.7823	0.8049	0.3480	8.00	
H14A	0.9827	0.6977	0.1910	6.41	
H14B	1.0763	0.6391	0.2774	6.41	
H14C	1.0392	0.7515	0.3378	6.41	

Table 3. Anisotropic displacement parameters
atom	U11	U22	U33	U12	U13	U23	
O1	0.0676(12)	0.0469(10)	0.129(2)	−0.0035(7)	−0.0046(13)	0.0140(11)	
O2	0.078(2)	0.0687(12)	0.105(2)	0.0198(10)	0.0118(13)	0.0182(12)	
N1	0.0629(13)	0.0412(9)	0.077(2)	0.0009(8)	−0.0048(11)	0.0032(10)	
N2	0.0643(12)	0.0423(9)	0.0669(13)	0.0003(8)	−0.0016(10)	0.0063(9)	
N3	0.088(3)	0.136(4)	0.175(5)	−0.003(2)	0.037(3)	0.019(4)	
C1	0.059(2)	0.0477(12)	0.075(2)	−0.0020(9)	−0.0117(13)	−0.0002(12)	
C2	0.058(2)	0.0501(12)	0.067(2)	0.0017(9)	−0.0089(12)	0.0075(11)	
C3	0.067(2)	0.0493(11)	0.061(2)	0.0053(10)	−0.0042(12)	0.0030(10)	
C4	0.063(2)	0.0534(13)	0.059(2)	0.0074(10)	0.0062(13)	0.0008(11)	
C5	0.063(2)	0.064(2)	0.074(2)	0.0026(11)	0.007(2)	0.0029(13)	
C6	0.071(2)	0.082(2)	0.091(3)	0.010(2)	0.009(2)	0.017(2)	
C7	0.084(2)	0.075(2)	0.108(3)	0.028(2)	0.027(3)	0.024(2)	
C8	0.123(3)	0.053(2)	0.082(2)	0.024(2)	0.029(3)	0.007(2)	
C9	0.100(3)	0.0532(13)	0.066(2)	0.0101(13)	0.008(2)	0.0013(12)	
C10	0.062(2)	0.063(2)	0.065(2)	0.0056(11)	-0.0059(13)	0.0111(13)	
C11	0.066(2)	0.069(2)	0.107(3)	0.0013(13)	0.004(2)	0.025(2)	
C12	0.073(3)	0.091(3)	0.106(3)	−0.003(2)	0.009(2)	0.023(2)	
C13	0.088(2)	0.050(2)	0.115(3)	0.0105(13)	0.012(2)	0.014(2)	
C14	0.072(2)	0.0608(13)	0.071(2)	-0.0065(12)	0.006(2)	0.0065(13)	
The general temperature factor expression: exp(-2p2(a*2U11h2 + b*2U22k2 + c*2U33l2 + 2a*b*U12hk + 2a*c*U13hl + 2b*c*U23kl)).
Table 4. Bond lengths (Å)
atom	atom	distance	atom	atom	distance	
O1	C1	1.231(3)	O2	C10	1.218(4)	
N1	N2	1.406(3)	N1	C1	1.404(4)	
N1	C4	1.422(4)	N2	C3	1.337(4)	
N2	C14	1.452(4)	N3	C12	1.141(6)	
C1	C2	1.442(4)	C2	C3	1.390(4)	
C2	C10	1.446(4)	C3	C13	1.492(4)	
C4	C5	1.385(4)	C4	C9	1.391(4)	
C5	C6	1.389(5)	C6	C7	1.373(6)	
C7	C8	1.380(6)	C8	C9	1.394(5)	
C10	C11	1.523(5)	C11	C12	1.423(6)	


Table 5. Bond lengths involving hydrogens (Å)
atom	atom	distance	atom	atom	distance	
C5	H5	0.93	C6	H6	0.93	
C7	H7	0.93	C8	H8	0.93	
C9	H9	0.93	C11	H11A	0.97	
C11	H11B	0.97	C13	H13A	0.96	
C13	H13B	0.96	C13	H13C	0.96	
C14	H14A	0.96	C14	H14B	0.96	
C14	H14C	0.96				
Table 6. Bond angles (°).
atom	atom	atom	angle		atom	atom	atom	angle	
N2	N1	C1	108.28(19)	N2	N1	C4	120.3(2)	
C1	N1	C4	123.8(2)		N1	N2	C3	108.9(2)	
N1	N2	C14	119.9(2)		C3	N2	C14	126.1(3)	
O1	C1	N1	122.1(3)		O1	C1	C2	132.6(3)	
N1	C1	C2	105.3(2)		C1	C2	C3	107.5(3)	
C1	C2	C10	126.2(3)		C3	C2	C10	126.2(3)	
N2	C3	C2	109.7(2)		N2	C3	C13	120.8(3)	
C2	C3	C13	129.5(3)		N1	C4	C5	120.5(3)	
N1	C4	C9	118.7(3)		C5	C4	C9	120.7(3)	
C4	C5	C6	120.0(3)		C5	C6	C7	119.5(4)	
C6	C7	C8	120.7(4)		C7	C8	C9	120.6(3)	
C4	C9	C8	118.4(3)		O2	C10	C2	125.0(3)	
O2	C10	C11	119.7(3)		C2	C10	C11	115.3(3)	
C10	C11	C12	114.2(3)		N3	C12	C11	178.0(5)	
Table 7. Bond angles involving hydrogens (°).
atom	atom	atom	angle	atom	atom	atom	angle	
C4	C5	H5	120	C6	C5	H5	120	
C5	C6	H6	120.2	C7	C6	H6	120.2	
C6	C7	H7	119.6	C8	C7	H7	119.7	
C7	C8	H8	119.7	C9	C8	H8	119.7	
C4	C9	H9	120.8	C8	C9	H9	120.8	
C10	C11	H11A	108.7	C10	C11	H11B	108.7	
C12	C11	H11A	108.7	C12	C11	H11B	108.7	
H11A	C11	H11B	107.6	C3	C13	H13A	109.5	
C3	C13	H13B	109.5	C3	C13	H13C	109.5	
H13A	C13	H13B	109.5	H13A	C13	H13C	109.5	
H13B	C13	H13C	109.5	N2	C14	H14A	109.5	
N2	C14	H14B	109.5	N2	C14	H14C	109.5	
H14A	C14	H14B	109.5	H14A	C14	H14C	109.5	
H14B	C14	H14C	109.5					


Table 8. Torsion Angles (°) (Those having bond angles > 160 or < 20 degrees are excluded.)
atom1	atom2	atom3	atom4	angle		atom1	atom2	atom3	atom4	angle	
N2	N1	C1	O1	−172.8(3) 	N2	N1	C1	C2	4.6(3) 	
C1	N1	N2	C3	−6.0(3) 		C1	N1	N2	C14	−162.3(2) 	
N2	N1	C4	C5	34.3(4) 		N2	N1	C4	C9	−148.9(3) 	
C4	N1	N2	C3	−156.7(3) 	C4	N1	N2	C14	47.0(4) 	
C1	N1	C4	C5	−111.8(3) 	C1	N1	C4	C9	65.1(4) 	
C4	N1	C1	O1	−23.4(5) 		C4	N1	C1	C2	154.1(3) 	
N1	N2	C3	C2	4.9(3) 		N1	N2	C3	C13	−174.8(2) 	
C14	N2	C3	C2	159.3(3) 		C14	N2	C3	C13	−20.3(4) 	
O1	C1	C2	C3	175.3(4) 		O1	C1	C2	C10	−2.1(6) 	
N1	C1	C2	C3	−1.8(3) 		N1	C1	C2	C10	−179.2(3) 	
C1	C2	C3	N2	−1.9(3) 		C1	C2	C3	C13	177.7(3) 	
C1	C2	C10	O2	−178.3(3) 	C1	C2	C10	C11	2.7(5) 	
C3	C2	C10	O2	4.7(5) 		C3	C2	C10	C11	−174.3(3) 	
C10	C2	C3	N2	175.5(3) 		C10	C2	C3	C13	−4.9(5) 	
N1	C4	C5	C6	175.7(3) 		N1	C4	C9	C8	−175.1(3) 	
C5	C4	C9	C8	1.7(5) 		C9	C4	C5	C6	−1.1(5) 	
C4	C5	C6	C7	0.6(5) 		C5	C6	C7	C8	−0.9(6) 	
C6	C7	C8	C9	1.6(6) 		C7	C8	C9	C4	−2.0(5) 	
O2	C10	C11	C12	5.0(5) 		C2	C10	C11	C12	−176.0(3) 	
Table 9. Intramolecular contacts less than 3.60 Å.
atom	atom	distance		atom	atom	distance	
O1	N2	3.447(3)		O1	C3	3.497(3)	
O1	C4	2.888(4)		O1	C9	3.069(5)	
O1	C10	3.140(4)		O1	C11	2.885(5)	
O2	N3	3.451(6)		O2	C3	2.969(4)	
O2	C12	2.723(5)		O2	C13	3.038(5)	
N2	C5	2.922(4)		N3	C10	3.491(6)	
C1	C5	3.503(4)		C1	C9	3.145(5)	
C1	C11	2.956(5)		C2	C4	3.595(4)	
C3	C4	3.535(4)		C4	C7	2.759(5)	
C4	C14	3.015(4)		C5	C8	2.763(5)	
C5	C14	3.199(5)		C6	C9	2.791(5)	
C10	C13	3.195(5)		C13	C14	2.991(5)	


Table 10. Intramolecular contacts less than 3.60 Å involving hydrogens
atom	atom	distance		atom	atom	distance	
O1	H9	2.843		O1	H11A	2.498	
O1	H11B	2.667		O2	H11A	3.011	
O2	H11B	2.963		O2	H13B	2.603	
O2	H13C	3.11		N1	H5	2.607	
N1	H9	2.589		N1	H14A	2.917	
N1	H14B	2.543		N1	H14C	3.207	
N2	H5	2.654		N2	H13A	2.526	
N2	H13B	3.144		N2	H13C	2.975	
N3	H11A	3.019		N3	H11B	3.008	
C1	H9	3.061		C1	H11A	2.783	
C1	H11B	2.913		C2	H11A	2.677	
C2	H11B	2.73		C2	H13A	3.332	
C2	H13B	2.8		C2	H13C	2.963	
C3	H14A	2.748		C3	H14B	3.24	
C3	H14C	2.752		C4	H6	3.243	
C4	H8	3.234		C4	H14A	3.597	
C4	H14B	2.603		C5	H7	3.226	
C5	H9	3.254		C5	H14B	2.652	
C5	H14C	3.591		C6	H8	3.227	
C7	H5	3.226		C7	H9	3.25	
C8	H6	3.232		C9	H5	3.25	
C9	H7	3.246		C10	H13B	3.012	
C10	H13C	3.397		C13	H14A	3.076	
C13	H14C	2.849		C14	H5	2.888	
C14	H13A	2.628		C14	H13C	3.353	
H5	H6	2.323		H5	H14B	2.503	
H5	H14C	3.052		H6	H7	2.302	
H7	H8	2.301		H8	H9	2.331	
H13A	H14A	2.79		H13A	H14B	3.542	
H13A	H14C	2.212		H13C	H14A	3.153	
H13C	H14C	3.32					
Table 11. Intermolecular contacts less than 3.60 Å
atom	atom	distance		atom	atom	distance	
O1	C71	3.331(4)		O1	C142	3.241(4)	
O2	C143	3.286(4)		N1	C54	3.411(5)	
N3	C105	3.510(7)		N3	C115	3.387(8)	
C5	N12	3.411(5)		C7	O16	3.331(4)	
C10	N37	3.510(7)		C11	N37	3.387(8)	
C14	O14	3.241(4)		C14	O28	3.286(4)	


Symmetry Operators:
(1) X + 1/2 − 1, −Y + 1/2, Z	(2) −X + 2, −Y + 1, Z + 1/2	
(3) X + 1/2 − 1, −Y + 1/2 + 1, Z	(4) −X + 2, −Y + 1, Z + 1/2 − 1	
(5) −X + 1, −Y + 1, Z + 1/2	(6) X + 1/2, −Y + 1/2, Z	
(7) −X + 1, −Y + 1, Z + 1/2 − 1	(8) X + 1/2, −Y + 1/2 + 1, Z	
Table 12. Intermolecular contacts less than 3.60 Å involving hydrogens
atom	atom	distance		atom	atom	distance	
O1	H71	2.568		O1	H81	3.286	
O1	H13C2	3.25		O1	H14A3	3.113	
O1	H14B3	2.548		O2	H54	3.052	
O2	H73	3.499		O2	H95	2.899	
O2	H13A4	3.347		O2	H14B4	3.451	
O2	H14C4	2.522		N1	H56	3.258	
N2	H93	3.481		N3	H67	2.74	
N3	H85	2.84		N3	H11A8	2.717	
N3	H13A2	3.498		N3	H13A4	3.356	
N3	H13B4	3.321		N3	H14C2	3.481	
C1	H66	3.555		C1	H14B3	3.137	
C2	H66	3.555		C3	H83	3.511	
C4	H14A3	2.983		C4	H14B3	3.471	
C5	H93	3.388		C5	H11B6	3.541	
C5	H13B9	3.215		C5	H13C9	3.479	
C5	H14A3	3.172		C6	H11B6	3.152	
C6	H13B9	3.284		C6	H14A3	3.221	
C7	H11B6	3.422		C7	H14A3	3.09	
C8	H11A10	3.407		C8	H13B6	3.387	
C8	H14A3	2.894		C9	H56	3.291	
C9	H14A3	2.864		C10	H14C4	3.225	
C11	H13A2	3.228		C11	H13C2	3.205	
C12	H67	3.513		C12	H85	3.529	
C12	H11A8	3.593		C12	H13A2	3.152	
C12	H13A4	3.461		C12	H14C2	3.593	
C13	H54	3.347		C13	H64	3.415	
C13	H83	3.426		C13	H11B11	2.901	
C13	H14B4	3.559		C14	H13C9	3.4	
H5	O29	3.052		H5	N13	3.258	
H5	C93	3.291		H5	C139	3.347	
H5	H93	2.65		H5	H13B9	2.689	
H5	H13C9	3.143		H6	N312	2.74	
H6	C13	3.555		H6	C23	3.555	
H6	C1212	3.513		H6	C139	3.415	
H6	H11B6	3.229		H6	H13A9	3.362	
H6	H13B9	2.833		H6	H13C9	3.538	
H7	O110	2.568		H7	O26	3.499	

Table 12. Cont.
atom	atom	distance		atom	atom	distance	
H7	H910	3.458		H7	H11A10	3.593	
H7	H13C3	3.357		H8	O110	3.286	
H8	N313	2.84		H8	C36	3.511	
H8	C1213	3.529		H8	C136	3.426	
H8	H11A10	2.84		H8	H13A6	3.564	
H8	H13B6	2.804		H8	H14A3	3.341	
H9	O213	2.899		H9	N26	3.481	
H9	C56	3.388		H9	H56	2.65	
H9	H71	3.458		H9	H14A3	3.321	
H11A	N314	2.717		H11A	C81	3.407	
H11A	C1214	3.593		H11A	H71	3.593	
H11A	H81	2.84		H11A	H13A2	3.443	
H11A	H13C2	3.23		H11B	C53	3.541	
H11B	C63	3.152		H11B	C73	3.422	
H11B	C132	2.901		H11B	H63	3.229	
H11B	H13A2	2.616		H11B	H13B2	3.285	
H11B	H13C2	2.415		H11B	H14B3	3.466	
H13A	O29	3.347		H13A	N311	3.498	
H13A	N39	3.356		H13A	C1111	3.228	
H13A	C1211	3.152		H13A	C129	3.461	
H13A	H64	3.362		H13A	H83	3.564	
H13A	H11A11	3.443		H13A	H11B11	2.616	
H13B	N39	3.321		H13B	C54	3.215	
H13B	C64	3.284		H13B	C83	3.387	
H13B	H54	2.689		H13B	H64	2.833	
H13B	H83	2.804		H13B	H11B11	3.285	
H13B	H14B4	3.533		H13C	O111	3.25	
H13C	C54	3.479		H13C	C1111	3.205	
H13C	C144	3.4		H13C	H54	3.143	
H13C	H64	3.538		H13C	H76	3.357	
H13C	H11A11	3.23		H13C	H11B11	2.415	
H13C	H14B4	2.763		H13C	H14C4	3.159	
H14A	O16	3.113		H14A	C46	2.983	
H14A	C56	3.172		H14A	C66	3.221	
H14A	C76	3.09		H14A	C86	2.894	
H14A	C96	2.864		H14A	H86	3.341	
H14A	H96	3.321		H14B	O16	2.548	
H14B	O29	3.451		H14B	C16	3.137	
H14B	C46	3.471		H14B	C139	3.559	
H14B	H11B6	3.466		H14B	H13B9	3.533	
H14B	H13C9	2.763		H14C	O29	2.522	
H14C	N311	3.481		H14C	C109	3.225	
H14C	C1211	3.593		H14C	H13C9	3.159	


Symmetry Operators:
(1) X + 1/2 − 1, −Y + 1/2, Z	(2) −X + 1/2 + 1, Y + 1/2 − 1, Z + 1/2	
(3) −X + 2, −Y + 1, Z + 1/2	(4) X + 1/2 − 1, −Y + 1/2 + 1, Z	
(5) −X + 1/2 + 1, Y + 1/2, Z + 1/2	(6) −X + 2, −Y + 1, Z + 1/2 − 1	
(7) X − 1, Y, Z	(8) –X + 1, −Y + 1, Z + 1/2	
(9) X + 1/2, −Y + 1/2 + 1, Z	(10) X + 1/2, −Y + 1/2, Z	
(11) −X + 1/2 + 1, Y + 1/2, Z + 1/2 − 1	(12) X + 1, Y, Z	
(13) –X + 1/2 + 1, Y + 1/2 − 1, Z + 1/2 − 1	(14) −X + 1, −Y + 1, Z + 1/2 − 1	
