# Supplementary material for: Studies on 3-Oxoalkanenitriles: Novel Rearrangement Reactions Observed in Studies of the Chemistry of 3-Heteroaroyl-3-Oxoalkanenitriles as Novel Routes to 2-Dialkylaminopyridines
Source: Molecules. 2012 Jan 18;17(1):897–909. doi: 10.3390/molecules17010897 (PMC6268551; doi:10.3390/molecules17010897)
Supplement: Supplementary file 1 [file molecules-17-00897-s001.zip › molecules-12690-supplementary/X Ray Data 1a and 5/X ray data for compound 5 CAMBzIm/Report 2/X-ray Structure Report.rtf]

X-ray Structure Report for 221210 
December 25, 2010 
Experimental 
Data Collection
A colorless prism crystal of C13H13N3O2 having approximate dimensions of 0.200 × 0.200 × 0.200 mm was mounted on a glass fiber. All measurements were made on a diffractometer Mo-Ka radiation.
Cell constants and an orientation matrix for data collection, obtained from a least-squares refinement using the setting angles of 4878 carefully centered reflections in the range 6.08 < 2q < 54.97° corresponded to a primitive monoclinic cell with dimensions:
a = 10.6950(9) Å
b = 8.4799(6) Å b = 89.978(5)°
c = 13.3940(12) Å
V = 1214.7(2) Å3
For Z = 4 and F.W. = 243.26, the calculated density is 1.330 g/cm3. The reflection conditions of:
h0l: h + l = 2n
0k0: k = 2n
uniquely determine the space group to be:
P21/n (#14) 
The data were collected at a temperature of 0 ± 1 °C using the w-2q scan technique to a maximum 2q value of 54.9°. Omega scans of several intense reflections, made prior to data collection, had an average width at half-height of 0.00o with a take-off angle of 6.0°. Scans of (0.00 + 0.00 tan q)° were made at a speed of 0.0°/min (in w).
Data Reduction
Of the 11565 reflections that were collected, 2764 were unique (Rint = 0.2168). No decay correction was applied. 
The linear absorption coefficient, m, for Mo-Ka radiation is 0.926 cm−1. The data were corrected for Lorentz and polarization effects. 
Structure Solution and Refinement
The structure was solved by charge flipping method and expanded using Fourier techniques. The non-hydrogen atoms were refined anisotropically. Hydrogen atoms were refined using the riding model. The final cycle of full-matrix least-squares refinement on F2 was based on 2764 observed reflections and 163 variable parameters and converged (largest parameter shift was 0.00 times its esd) with unweighted and weighted agreement factors of: 
R1 = S ||Fo| − |Fc|| / S |Fo| = 0.1110 
wR2 = [S(w (Fo2 − Fc2)2)/ S w(Fo2)2]1/2 = 0.3490
The standard deviation of an observation of unit weight was 1.21. Unit weights were used. The maximum and minimum peaks on the final difference Fourier map corresponded to 0.53 and −0.42 e−/Å3, respectively.
Neutral atom scattering factors were taken from Cromer and Waber. Anomalous dispersion effects were included in Fcalc; the values for Df' and Df" were those of Creagh and McAuley. The values for the mass attenuation coefficients are those of Creagh and Hubbell. All calculations were performed using the CrystalStructure crystallographic software package except for refinement, which was performed using SHELXL-97.
Experimental Details 
A. Crystal Data 
Empirical Formula	C13H13N3O2	
Formula Weight	243.26	
Crystal Color, Habit	colorless, prism	
Crystal Dimensions	0.200 × 0.200 × 0.200 mm	
Crystal System	monoclinic	
Lattice Type	Primitive	
No. of Reflections Used for Unit
Cell Determination (2q range)	4878 ( 6.1–55.0°)	
Omega Scan Peak Width at Half-height	0.00°	
Lattice Parameters	a = 10.6950(9) Å
b = 8.4799(6) Å
c = 13.3940(12) Å
b = 89.978(5)°
V = 1214.7(2) Å3	
Space Group	P21/n (#14)	
Z value	4	
Dcalc	1.330 g/cm3	
F000	512.00	
m(MoKa)	0.926 cm−1	

B. Intensity Measurements 
Diffractometer		
Radiation	MoKa (l = 0.71075 Å)	
Take-off Angle	2.8°	
Detector Aperture	2.0–2.5 mm horizontal
2.0 mm vertical	
Crystal to Detector Distance	21 mm	
Voltage, Current	50kV, 24mA	
Temperature	0.0 °C	
Scan Type	w-2q	
Scan Rate	0.0°/min (in w) (up to 0 scans)	
Scan Width	(0.00 + 0.00 tan q)°	
2qmax	54.9°	
No. of Reflections Measured	Total: 11565
Unique: 2764 (Rint = 0.2168)	
Corrections	Lorentz-polarization	
C. Structure Solution and Refinement 
Structure Solution	Charge Flipping (Superflip)	
Refinement	Full-matrix least-squares on F2	
Function Minimized	S w (Fo2 − Fc2)2	
Least Squares Weights	w = 1/ [ s2 (Fo2) + (0.1636P)2 + 0.0000P] where P = (Max(Fo2,0) + 2Fc2)/3	
2qmax cutoff	54.9°	
Anomalous Dispersion	All non-hydrogen atoms	
No. Observations (All reflections)	2764	
No. Variables	163	
Reflection/Parameter Ratio	16.96	
Residuals: R1 (I > 2.00s(I))	0.1110	
Residuals: R (All reflections)	0.1401	
Residuals: wR2 (All reflections)	0.3490	
Goodness of Fit Indicator	1.213	
Max Shift/Error in Final Cycle	0.000	
Maximum peak in Final Diff. Map	0.53 e−/Å3	
Minimum peak in Final Diff. Map	−0.42 e−/Å3	

Table 1. Atomic coordinates and Biso/Beq.
atom	x	y	z	Beq	
O1	0.9800(3)	0.4473(3)	0.8757(2)	4.77(6)	
O2	0.5199(3)	0.4473(3)	0.8754(2)	4.78(6)	
N1	0.8565(3)	0.2343(3)	0.8871(2)	3.37(6)	
N2	0.6435(3)	0.2355(3)	0.8869(2)	3.37(6)	
N3	0.7504(5)	0.1508(6)	1.1353(3)	6.98(11)	
C1	0.7488(3)	0.3369(4)	0.9086(3)	3.35(6)	
C2	0.8153(3)	0.0774(4)	0.8793(3)	3.48(7)	
C3	0.8820(4)	−0.0632(4)	0.8770(3)	4.43(8)	
C4	0.8141(5)	−0.2027(4)	0.8744(3)	5.03(9)	
C5	0.6847(5)	−0.2029(5)	0.8738(3)	4.97(9)	
C6	0.6201(4)	−0.0633(4)	0.8781(3)	4.32(8)	
C7	0.6849(3)	0.0767(4)	0.8795(3)	3.31(6)	
C8	0.9694(3)	0.3050(4)	0.8666(3)	3.62(6)	
C9	1.0757(4)	0.2021(5)	0.8329(4)	5.22(9)	
C10	0.5305(3)	0.3048(4)	0.8670(3)	3.64(7)	
C11	0.4255(4)	0.2033(5)	0.8338(4)	5.43(9)	
C12	0.7501(3)	0.3949(4)	1.0173(3)	3.98(7)	
C13	0.7503(4)	0.2605(5)	1.0852(3)	4.82(8)	
Beq = 8/3 p2 (U11(aa*)2 + U22(bb*)2 + U33(cc*)2 + 2U12(aa*bb*)cos g + 2U13(aa*cc*)cos b + 2U23(bb*cc*)cos a).
Table 2. Atomic coordinates and Biso involving hydrogen atoms.
atom	x	y	z	Biso	
H1	0.7489	0.4273	0.8630	4.02	
H3	0.9689	−0.0638	0.8772	5.32	
H4	0.8566	−0.2983	0.8730	6.04	
H5	0.6414	−0.2979	0.8706	5.96	
H6	0.5332	−0.0634	0.8799	5.18	
H9A	1.0474	0.1329	0.7809	6.26	
H9B	1.1053	0.1406	0.8883	6.26	
H9C	1.1424	0.2669	0.8081	6.26	
H11A	0.4559	0.1260	0.7874	6.52	
H11B	0.3630	0.2671	0.8020	6.52	
H11C	0.3895	0.1511	0.8906	6.52	
H12A	0.8239	0.4588	1.0288	4.78	
H12B	0.6770	0.4597	1.0298	4.78	

Table 3. Anisotropic displacement parameters.
atom	U11	U22	U33	U12	U13	U23	
O1 	0.054(2)	0.050(2)	0.078(2)	−0.0109(10)	0.0129(12)	0.0006(12)	
O2 	0.058(2)	0.049(2)	0.075(2)	0.0110(10)	0.0090(13)	−0.0014(12)	
N1 	0.047(2)	0.033(2)	0.048(2)	0.0014(10)	0.0114(11)	−0.0012(10)	
N2 	0.045(2)	0.034(2)	0.050(2)	−0.0017(10)	0.0064(11)	-0.0006(10)	
N3 	0.131(4)	0.078(3)	0.057(3)	0.005(3)	0.008(2)	0.000(2)	
C1 	0.040(2)	0.030(2)	0.057(2)	0.0009(11)	0.0083(12)	−0.0025(12)	
C2 	0.062(2)	0.035(2)	0.035(2)	0.0009(12)	0.0117(13)	0.0030(11)	
C3 	0.068(3)	0.042(2)	0.058(3)	0.011(2)	0.017(2)	0.001(2)	
C4 	0.097(3)	0.035(2)	0.060(3)	0.008(2)	0.015(2)	−0.003(2)	
C5 	0.083(3)	0.039(2)	0.067(3)	−0.009(2)	0.002(2)	0.000(2)	
C6 	0.066(3)	0.042(2)	0.056(2)	−0.011(2)	0.002(2)	−0.001(2)	
C7 	0.053(2)	0.033(2)	0.040(2)	−0.0011(11)	0.0076(13)	0.0008(11)	
C8 	0.045(2)	0.047(2)	0.045(2)	−0.0053(13)	0.0112(13)	0.0004(13)	
C9 	0.052(2)	0.066(3)	0.080(3)	−0.000(2)	0.024(2)	0.005(2)	
C10 	0.047(2)	0.048(2)	0.044(2)	0.0049(13)	0.0095(13)	0.0042(13)	
C11 	0.056(2)	0.067(3)	0.084(3)	−0.001(2)	0.000(2)	0.000(2)	
C12 	0.052(2)	0.038(2)	0.061(2)	−0.0008(13)	0.009(2)	−0.012(2)	
C13 	0.068(3)	0.065(3)	0.050(2)	−0.003(2)	0.010(2)	−0.017(2)	
The general temperature factor expression: exp(-2p2 (a*2U11h2 + b*2U22k2 + c*2U33l2 + 2a*b*U12hk + 2a*c*U13hl + 2b*c*U23kl)).
Table 4. Bond lengths (Å).
atom	atom	distance		atom	atom	distance	
O1	C8	1.218(4)		O2	C10	1.219(4)	
N1	C1	1.472(4)		N1	C2	1.406(4)	
N1	C8	1.376(4)		N2	C1	1.447(4)	
N2	C7	1.421(4)		N2	C10	1.369(4)	
N3	C13	1.147(6)		C1	C12	1.537(5)	
C2	C3	1.390(5)		C2	C7	1.394(5)	
C3	C4	1.388(6)		C4	C5	1.384(7)	
C5	C6	1.372(6)		C6	C7	1.375(5)	
C8	C9	1.502(5)		C10	C11	1.484(6)	
C12	C13	1.458(6)					
Table 5. Bond lengths involving hydrogens (Å)
atom	atom	distance		atom	atom	distance	
C1	H1	0.98		C3	H3	0.93	
C4	H4	0.93		C5	H5	0.93	
C6	H6	0.93		C9	H9A	0.96	
C9	H9B	0.96		C9	H9C	0.96	
C11	H11A	0.96		C11	H11B	0.96	
C11	H11C	0.96		C12	H12A	0.97	
C12	H12B	0.97					
Table 6. Bond angles (°).
atom	atom	atom	angle		atom	atom	atom	angle	
C1	N1	C2	109.2(3)		C1	N1	C8	117.9(3)	
C2	N1	C8	132.2(3)		C1	N2	C7	109.5(3)	
C1	N2	C10	118.1(3)		C7	N2	C10	131.9(3)	
N1	C1	N2	102.6(3)		N1	C1	C12	111.5(3)	
N2	C1	C12	112.8(3)		N1	C2	C3	130.8(4)	
N1	C2	C7	108.5(3)		C3	C2	C7	120.7(3)	
C2	C3	C4	117.6(4)		C3	C4	C5	121.6(4)	
C4	C5	C6	120.2(4)		C5	C6	C7	119.5(4)	
N2	C7	C2	108.0(3)		N2	C7	C6	131.5(3)	
C2	C7	C6	120.5(3)		O1	C8	N1	119.6(3)	
O1	C8	C9	122.3(3)		N1	C8	C9	118.1(3)	
O2	C10	N2	119.3(3)		O2	C10	C11	122.2(3)	
N2	C10	C11	118.5(3)		C1	C12	C13	110.0(3)	
N3	C13	C12	177.2(5)						
Table 7. Bond angles involving hydrogens (°).
atom	atom	atom	angle		atom	atom	atom	angle	
N1	C1	H1	109.9		N2	C1	H1	109.9	
C12	C1	H1	109.9		C2	C3	H3	121.2	
C4	C3	H3	121.2		C3	C4	H4	119.2	
C5	C4	H4	119.2		C4	C5	H5	119.9	
C6	C5	H5	119.9		C5	C6	H6	120.3	
C7	C6	H6	120.3		C8	C9	H9A	109.5	
C8	C9	H9B	109.5		C8	C9	H9C	109.5	
H9A	C9	H9B	109.5		H9A	C9	H9C	109.5	
H9B	C9	H9C	109.5		C10	C11	H11A	109.5	
C10	C11	H11B	109.5		C10	C11	H11C	109.5	
H11A	C11	H11B	109.5		H11A	C11	H11C	109.5	
H11B	C11	H11C	109.5		C1	C12	H12A	109.7	
C1	C12	H12B	109.7		C13	C12	H12A	109.7	
C13	C12	H12B	109.7		H12A	C12	H12B	108.2	

Table 8. Torsion Angles (°) (Those having bond angles > 160 or < 20 degrees are excluded).
atom1	atom2	atom3	atom4	angle		atom1	atom2	atom3	atom4	angle	
C1	N1	C2	C3	−167.5(3) 	C1	N1	C2	C7	8.8(3) 	
C2	N1	C1	N2	−14.1(3) 		C2	N1	C1	C12	107.0(3) 	
C1	N1	C8	O1	6.7(4) 		C1	N1	C8	C9	−172.9(3) 	
C8	N1	C1	N2	158.0(3) 		C8	N1	C1	C12	−80.9(3) 	
C2	N1	C8	O1	176.7(3) 		C2	N1	C8	C9	−3.0(5) 	
C8	N1	C2	C3	21.9(6) 		C8	N1	C2	C7	−161.8(3) 	
C1	N2	C7	C2	−9.8(3) 		C1	N2	C7	C6	166.7(3) 	
C7	N2	C1	N1	14.4(3) 		C7	N2	C1	C12	−105.8(3) 	
C1	N2	C10	O2	−5.4(4) 		C1	N2	C10	C11	173.4(3) 	
C10	N2	C1	N1	−158.9(3) 	C10	N2	C1	C12	81.0(3) 	
C7	N2	C10	O2	−176.8(3) 	C7	N2	C10	C11	1.9(5) 	
C10	N2	C7	C2	162.2(3) 		C10	N2	C7	C6	−21.3(6) 	
N1	C1	C12	C13	−57.7(3) 		N2	C1	C12	C13	57.1(3) 	
N1	C2	C3	C4	176.3(3) 		N1	C2	C7	N2	0.5(3) 	
N1	C2	C7	C6	−176.4(3) 	C3	C2	C7	N2	177.3(3) 	
C3	C2	C7	C6	0.3(5) 		C7	C2	C3	C4	0.4(5) 	
C2	C3	C4	C5	0.2(6) 		C3	C4	C5	C6	−1.4(6) 	
C4	C5	C6	C7	2.1(6) 		C5	C6	C7	N2	−177.7(3) 	
C5	C6	C7	C2	-1.6(5) 							
Table 9. Intramolecular contacts less than 3.60 Å.
atom	atom	distance		atom	atom	distance	
O1	C1	2.681(4)		O1	C2	3.598(4)	
O1	C12	3.137(5)		O2	C1	2.658(4)	
O2	C12	3.142(5)		N1	N3	3.582(5)	
N1	C6	3.575(5)		N1	C10	3.547(4)	
N1	C13	2.894(5)		N2	N3	3.592(5)	
N2	C3	3.597(5)		N2	C8	3.546(4)	
N2	C13	2.900(5)		N3	C1	3.422(5)	
N3	C2	3.553(5)		N3	C7	3.553(5)	
C2	C5	2.758(5)		C2	C9	3.043(5)	
C2	C12	3.339(5)		C2	C13	3.241(5)	
C3	C6	2.801(6)		C3	C8	3.262(5)	
C3	C9	3.114(6)		C4	C7	2.744(5)	
C6	C10	3.269(5)		C6	C11	3.129(6)	
C7	C11	3.037(5)		C7	C12	3.342(5)	
C7	C13	3.242(5)		C8	C12	3.187(5)	
C10	C12	3.187(5)					

Table 10. Intramolecular contacts less than 3.60 Å involving hydrogens.
atom	atom	distance		atom	atom	distance	
O1	H1	2.484		O1	H9A	3.039	
O1	H9B	2.93		O1	H9C	2.484	
O1	H12A	2.646		O2	H1	2.46	
O2	H11A	3.047		O2	H11B	2.474	
O2	H11C	2.88		O2	H12B	2.667	
N1	H3	2.803		N1	H9A	2.632	
N1	H9B	2.777		N1	H9C	3.247	
N1	H12A	2.71		N1	H12B	3.314	
N2	H6	2.797		N2	H11A	2.581	
N2	H11B	3.22		N2	H11C	2.809	
N2	H12A	3.305		N2	H12B	2.721	
N3	H12A	3.078		N3	H12B	3.078	
C2	H1	3.059		C2	H4	3.218	
C2	H6	3.245		C2	H9A	2.849	
C2	H9B	3.15		C3	H5	3.254	
C3	H9A	2.747		C3	H9B	2.952	
C4	H6	3.229		C5	H3	3.261	
C6	H4	3.221		C6	H11A	2.671	
C6	H11C	3.068		C7	H1	3.059	
C7	H3	3.263		C7	H5	3.213	
C7	H11A	2.774		C7	H11C	3.224	
C8	H1	2.577		C8	H3	3.13	
C8	H12A	2.973		C9	H3	2.596	
C10	H1	2.557		C10	H6	3.127	
C10	H12B	2.989		C11	H6	2.612	
C13	H1	3.296		H1	H12A	2.377	
H1	H12B	2.378		H3	H4	2.324	
H3	H9A	2.269		H3	H9B	2.27	
H3	H9C	3.487		H4	H5	2.302	
H5	H6	2.304		H6	H11A	2.19	
H6	H11B	3.501		H6	H11C	2.385	

Table 11. Intermolecular contacts less than 3.60 Å.
atom	atom	distance		atom	atom	distance	
O1	O11	3.475(4)		O1	C42	3.459(5)	
O1	C63	3.564(5)		O1	C121	3.490(5)	
O2	O24	3.482(4)		O2	C33	3.540(5)	
O2	C52	3.450(5)		O2	C124	3.492(5)	
N1	C53	3.563(5)		N2	C43	3.567(5)	
N3	C95	3.549(6)		N3	C96	3.472(6)	
N3	C117	3.567(7)		N3	C118	3.480(6)	
C3	O29	3.540(5)		C3	C109	3.578(5)	
C4	O110	3.459(5)		C4	N29	3.567(5)	
C5	O210	3.450(5)		C5	N19	3.563(5)	
C6	O19	3.564(5)		C6	C89	3.592(5)	
C8	C63	3.592(5)		C9	N35	3.549(6)	
C9	N311	3.472(6)		C10	C33	3.578(5)	
C11	N37	3.567(7)		C11	N312	3.480(6)	
C12	O11	3.490(5)		C12	O24	3.492(5)	
Symmetry Operators
(1) −X + 2, −Y + 1, −Z + 2	(2) X, Y + 1, Z	
(3) −X + 1/2 + 1, Y + 1/2, −Z + 1/2 + 1	(4) −X + 1, −Y + 1, −Z + 2	
(5) −X + 2, −Y, −Z + 2	(6) X + 1/2 − 1, −Y + 1/2, Z + 1/2	
(7) −X + 1, −Y, −Z + 2	(8) X + 1/2, −Y + 1/2, Z + 1/2	
(9) −X + 1/2 + 1, Y + 1/2 − 1, −Z + 1/2 + 1	(10) X, Y − 1, Z	
(11) X + 1/2, −Y + 1/2, Z + 1/2 − 1	(12) X + 1/2 − 1, −Y + 1/2, Z + 1/2 − 1	
Table 12. Intermolecular contacts less than 3.60 Å involving hydrogens.
atom	atom	distance		atom	atom	distance	
O1	H41	2.529		O1	H62	3.427	
O1	H11A2	2.745		O1	H12A3	2.583	
O2	H32	3.386		O2	H51	2.522	
O2	H9A2	2.716		O2	H12B4	2.582	
N1	H52	3.464		N2	H42	3.492	
N3	H35	3.095		N3	H66	3.129	
N3	H9A5	3.425		N3	H9A7	3.447	
N3	H9B5	2.93		N3	H9C7	2.679	
N3	H11A6	3.384		N3	H11A8	3.545	
N3	H11B8	2.63		N3	H11C6	2.986	
C1	H41	3.335		C1	H51	3.342	
C2	H19	3.552		C2	H52	3.54	
C3	H19	3.507		C3	H9B5	3.215	
C4	H110	3.218		C4	H19	3.432	
C4	H9B5	3.336		C4	H11A9	3.585	
C4	H12A10	3.54		C5	H110	3.213	
C5	H19	3.433		C5	H9A9	3.521	
C5	H11C6	3.283		C5	H12B10	3.544	

Table 12. Cont.
atom	atom	distance		atom	atom	distance	
C6	H19	3.52		C6	H11C6	3.189	
C7	H19	3.558		C7	H42	3.572	
C8	H41	3.575		C8	H52	3.502	
C8	H62	3.485		C8	H11A2	3.507	
C8	H12A3	3.296		C9	H52	3.582	
C9	H11B11	3.149		C9	H11C11	3.471	
C9	H12A3	3.585		C10	H32	3.455	
C10	H42	3.542		C10	H51	3.572	
C10	H9A2	3.516		C10	H12B4	3.29	
C11	H9B12	3.541		C11	H9C12	3.095	
C11	H12B4	3.565		C12	H41	3.436	
C12	H51	3.464		C13	H35	3.472	
C13	H66	3.493		C13	H9A7	3.52	
C13	H9C7	3.209		C13	H11B8	3.152	
H1	C22	3.552		H1	C32	3.507	
H1	C41	3.218		H1	C42	3.432	
H1	C51	3.213		H1	C52	3.433	
H1	C62	3.52		H1	C72	3.558	
H1	H41	2.6		H1	H51	2.6	
H3	O29	3.386		H3	N35	3.095	
H3	C109	3.455		H3	C135	3.472	
H3	H35	3.527		H3	H9B5	3.305	
H3	H11A9	3.525		H3	H11B9	3.323	
H4	O110	2.529		H4	N29	3.492	
H4	C110	3.335		H4	C79	3.572	
H4	C810	3.575		H4	C109	3.542	
H4	C1210	3.436		H4	H110	2.6	
H4	H9B5	3.49		H4	H11A9	3.007	
H4	H12A10	2.953		H4	H12B10	3.509	
H5	O210	2.522		H5	N19	3.464	
H5	C110	3.342		H5	C29	3.54	
H5	C89	3.502		H5	C99	3.582	
H5	C1010	3.572		H5	C1210	3.464	
H5	H110	2.6		H5	H9A9	2.923	
H5	H11C6	3.448		H5	H12A10	3.543	
H5	H12B10	2.986		H6	O19	3.427	
H6	N36	3.129		H6	C89	3.485	
H6	C136	3.493		H6	H66	3.466	
H6	H9A9	3.466		H6	H9C9	3.455	
H6	H11C6	3.27		H9A	O29	2.716	

Table 12. Cont.
H9A	N35	3.425		H9A	N313	3.447	
H9A	C52	3.521		H9A	C109	3.516	
H9A	C1313	3.52		H9A	H52	2.923	
H9A	H62	3.466		H9A	H11B11	3.573	
H9A	H11B9	3.432		H9B	N35	2.93	
H9B	C35	3.215		H9B	C45	3.336	
H9B	C1111	3.541		H9B	H35	3.305	
H9B	H45	3.49		H9B	H11B11	3.175	
H9B	H11C11	3.041		H9C	N313	2.679	
H9C	C1111	3.095		H9C	C1313	3.209	
H9C	H62	3.455		H9C	H11A11	3.571	
H9C	H11A2	3.466		H9C	H11B11	2.361	
H9C	H11C11	3.029		H9C	H12A3	3.211	
H11A	O19	2.745		H11A	N36	3.384	
H11A	N314	3.545		H11A	C42	3.585	
H11A	C89	3.507		H11A	H32	3.525	
H11A	H42	3.007		H11A	H9C12	3.571	
H11A	H9C9	3.466		H11B	N314	2.63	
H11B	C912	3.149		H11B	C1314	3.152	
H11B	H32	3.323		H11B	H9A12	3.573	
H11B	H9A2	3.432		H11B	H9B12	3.175	
H11B	H9C12	2.361		H11B	H12B4	3.261	
H11C	N36	2.986		H11C	C56	3.283	
H11C	C66	3.189		H11C	C912	3.471	
H11C	H56	3.448		H11C	H66	3.27	
H11C	H9B12	3.041		H11C	H9C12	3.029	
H11C	H12B4	3.541		H12A	O13	2.583	
H12A	C41	3.54		H12A	C83	3.296	
H12A	C93	3.585		H12A	H41	2.953	
H12A	H51	3.543		H12A	H9C3	3.211	
H12B	O24	2.582		H12B	C51	3.544	
H12B	C104	3.29		H12B	C114	3.565	
H12B	H41	3.509		H12B	H51	2.986	
H12B	H11B4	3.261		H12B	H11C4	3.541	


Symmetry Operators:
(1) X, Y + 1, Z	(2) –X + 1/2 + 1, Y + 1/2, −Z + 1/2 + 1	
(3) −X + 2, −Y + 1, −Z + 2	(4) –X + 1, −Y + 1, −Z + 2	
(5) −X + 2, −Y, −Z + 2	(6) −X + 1, −Y, −Z + 2	
(7) X + 1/2 − 1, −Y + 1/2, Z + 1/2	(8) X + 1/2, −Y + 1/2, Z + 1/2	
(9) −X + 1/2 + 1, Y + 1/2 − 1, −Z + 1/2 + 1	(10) X, Y − 1, Z	
(11) X + 1, Y, Z	(12) X − 1, Y, Z	
(13) X + 1/2, −Y + 1/2, Z + 1/2 − 1	(14) X + 1/2 − 1, −Y + 1/2, Z + 1/2 − 1	
