# Supplementary material for: Studies on 3-Oxoalkanenitriles: Novel Rearrangement Reactions Observed in Studies of the Chemistry of 3-Heteroaroyl-3-Oxoalkanenitriles as Novel Routes to 2-Dialkylaminopyridines
Source: Molecules. 2012 Jan 18;17(1):897–909. doi: 10.3390/molecules17010897 (PMC6268551; doi:10.3390/molecules17010897)
Supplement: Supplementary file 1 [file molecules-17-00897-s001.zip › molecules-12690-supplementary/X Ray Data 1a and 5/X ray data for compound 1a CAAP/Figure 1.doc]

Figure 1
